# Scientific Clinical Trial Publication Productivity in Medical Oncology: A Comparative Analysis and Data Review of Northern and Southern Italy

**DOI:** 10.3390/clinpract16090159

**Published:** 2026-08-27

**Authors:** Vittorio Gebbia, Sergio Rizzo, Dario Piazza, Daniela Sambataro, Giuseppa Scandurra, Maria Rosaria Valerio

**Affiliations:** 1 Department of Medicine and Surgery, Kore University of Enna, 94100 Enna, Italy; 2 Medical Oncology Unit, Mediterranean Institute for Transplantation and Advanced Specialized Therapies (IRCCS ISMETT), 90127 Palermo, Italy; sergio.rizzo@ismett.edu (S.R.); daniela.sambataro@unikore.it (D.S.); giuseppa.scandurra@unikore.it (G.S.); 3 Data Manager Unit, CdC Torina, 90100 Palermo, Italy; 4 Medical Oncology Unit, Ospedale Umberto I, 94100 Enna, Italy; 5 Medical Oncology Unit, Ospedale Cannizzaro, 95126 Catania, Italy; 6 Medical Oncology Unit, Policlinico P. Giaccone, Università di Palermo, 90127 Palermo, Italy; mariarosaria.valerio@unipa.it

**Keywords:** medical oncology, scientific productivity, regional divide, bibliometrics, impact factor, organization, institutions

## Abstract

**Background:** Italy exhibits a socioeconomic divide between its northern and southern regions, which extends into healthcare delivery and biomedical research. Medical oncology is particularly affected by this structural imbalance. This paper examines geographic disparities in clinical trial scientific publication output, citation impact, research funding, and collaboration networks in medical oncology between northern and southern Italy. **Methods:** A narrative bibliometric analysis was conducted using data from PubMed and other institutional databases of Italian Scientific Institutes for Hospitalization and Care. Data from the Italian National Agency for the Evaluation of Universities and Research Institutes, the Alliance Against Cancer, and the Italian Association for Cancer Research were also reviewed. Regional output was compared across two macro-areas, namely north and south Italy. **Results:** Northern Italian institutions account for a disproportionate share of publications, high-impact citations, and funded research projects. Northern Italy produces an estimated four to six times more medical oncology papers than southern Italy, driven by a higher concentration of cancer institutes, better-funded university hospitals, and stronger multidisciplinary infrastructure than in the south. Clinical trial density is highest in the north compared to the south (0.53 versus 0.02 per 100,000 inhabitants). **Conclusions:** The north–south divide in Italian oncology research productivity is real, multi-factorial, and persistent. The analysis reveals a substantial gap in research productivity, institutional capacity, and access to clinical trials between the two macro-regions. Targeted policy interventions, including decentralized trial models, dedicated funding streams for southern institutions, and stronger infrastructure for inter-regional collaboration, are urgently needed to ensure representativeness in oncology evidence generation and equitable scientific progress.

## 1. Introduction

Clinical research is crucial to delivering higher-quality, up-to-date, and more equitable care to cancer patients [1]. Medical oncology is especially important in this context, as it has become a major area of biomedical research driven by the high cancer burden, increased investment in translational science, and rapid technological advances that occurred in the last two decades. Research does not merely serve as a tool for scientific progress; it also profoundly influences healthcare [2]. In general, patients treated in research-active institutions tend to have better outcomes than those treated outside these settings [3,4,5,6].

Worldwide, effective cancer care and research depend on a system comprising specialized clinical centers, active clinical trial programs, robust research funding, and strong collaboration networks [7]. Gaps in any of these areas result in differences in the quality of evidence, which, in turn, may affect clinical guidelines, access to new treatments, and clinical outcomes. Research-active centers may offer highly specialized cancer care, innovative treatments, and access to cutting-edge clinical trials [8,9,10,11]. However, in most parts of the world, including some of the highly industrialized Western countries, the cancer research landscape remains suboptimal even to date [12]. In 2023, the Lancet Oncology European Ground Shot Commission reported updated, detailed data on patient-centered cancer research activity across Europe [12]. The findings reported a gap in cancer research activity between the lower-performing countries of Central/Eastern Europe and the rest of the European continent. Moreover, data showed that research goals were overemphasized. In contrast, other important domains, such as basic science, biopharmaceutical research, prevention, and early diagnosis, were understudied, underscoring the need for more coordinated, information- and data-driven cancer research strategies and planning [12].

The association between cancer research and patients’ clinical outcomes has also been reported in low-income countries [13,14,15]. A study in South America reported a statistically significant inverse linear correlation between rising scientific productivity and declining overall cancer mortality (*p* < 0.001), despite outputs being predominantly descriptive, mono-institutional, and of low international impact [15]. This correlation is also present in other areas of medicine, not just oncology. Shanian et al. explored the association between hospital research publications and clinical quality among US acute care hospitals [3]. This cross-sectional study showed that only 647 of 1604 study hospitals (40.3%) had ≥1 publication, except for the Council of Teaching Hospitals and Health Systems hospitals, which had significantly higher scientific output and citation rates than others. In multivariate analysis, bibliometric indicators were significantly correlated with teaching volume, number of beds, and not-for-profit or government status, and, most importantly, publications were associated with significantly lower risk-adjusted mortality rates for cardiovascular and respiratory diseases [3].

In this view, the establishment of Comprehensive Cancer Centers (CCC) in the Western world plays a pivotal role in achieving high-quality, networked infrastructures to reach the above-reported needs and thus increase the cure rate, improve patients’ survival and quality of life, and deal with research and care inequalities existing among diverse countries [16,17]. In the USA, the National Cancer Institute supports designated cancer research centers that are deeply involved in research and provide services directly to cancer patients [18]. According to the Organization of European Cancer Institutes (OECI), a CCC is “an organizational entity with a clear central governance spanning cancer care, research, and education generally in one geographical location [16,19]. CCC include a direct provision of an extensive range of high-quality cancer diagnostics and care covering at least all the major cancers; a high level of infrastructure, expertise, and innovation in cancer research, especially in translational and clinical research including early clinical trial units, but also in many cases basic science and discovery science; a university partnership as part of the center; or strong links with universities and research institutes; extensive international networking in research and clinical trials; and educational programs for clinicians, researchers, and patients” [16,19]. The number of published papers, country population, and gross domestic product showed no significant differences; however, the mean impact factor differed substantially [20].

Cancer is the second leading cause of death in Italy, accounting for about 28% of all deaths and around 390,000 new cases each year [21]. Italy is known worldwide for its contributions to biomedical research [22]. Its network of universities, research hospitals, and cancer centers has enabled Italian researchers to publish in top oncology journals and play key roles in major clinical trials [22,23]. However, a long-standing divide between northern and southern Italy affects many aspects of society, including academic medicine [24,25]. This divide began after Italy’s unification and has grown over time due to differences in industry, education, and infrastructure. Southern regions such as Campania, Calabria, Puglia, Basilicata, Molise, Sicily, and Sardinia account for about a third of Italy’s population but produce lower quality care and much less scientific research [26,27]. This gap may also include medical oncology.

While many studies have examined differences in cancer care and survival across Italy, less attention has been paid to a major root cause such as differences in scientific research productivity [27,28,29]. Understanding what drives research output in medical oncology is important for shaping policy, using resources wisely, and supporting fair international collaboration. This paper aims to reduce that gap by examining publication productivity in medical oncology across Italian regions, focusing on four main areas: total publication output, citation impact and quality, research funding and resources, and alliance networks and international visibility.

## 2. Materials and Methods

### 2.1. Study Design

This narrative–analytical bibliometric review examines differences in medical oncology research output between northern and southern Italy by analyzing clinical trial publications from January 2021 to March 2026. The analysis focused on published clinical trials, which are considered the most reliable measure because trial participation strongly indicates research activity and institutional capacity [30,31]. We included studies and institutional reports on medical oncology research productivity, bibliometric indicators, clinical trial enrollment, and research funding in Italy.

### 2.2. Data Sources and Search

We classified publications by geographic region based on institutional affiliation. To do so, we grouped Italian Nomenclature of Territorial Units for Statistics (NUTS-2) administrative regions into two main areas, following guidelines from the Italian National Institute of Statistics (ISTAT). The north includes Liguria, Lombardy, Piedmont, Val d’Aosta, Emilia-Romagna, Friuli-Venezia Giulia, Trentino-Alto Adige, and Veneto. The south includes Basilicata, Calabria, Campania, Molise, Puglia, Sardinia, and Sicily. Precise national bibliometric databases disaggregated by Italian region are not publicly available in a single repository. Given the heterogeneity of available data sources and the absence of a single consolidated database of regional Italian oncology publications, a multi-source approach was employed to estimate the number of medical oncology publications. The estimates are derived from annual reports of Istituti di Ricovero e Cura a Carattere Scintifico (IRCCS), PubMed affiliation data, and published bibliometric analyses of Italian cancer institutions. We searched PubMed on 24 March 2026 for oncology papers from northern and southern Italy using the PubMed E-utilities API. Query 1 (MeSH-based) uses PubMed’s controlled medical vocabulary, which is more precise but depends on NCBI curators’ correct indexing. Some recent papers from 2025–2026 may not yet be fully indexed. Query 2 (geographic-specific) targets northern or southern Italian cities and regions by affiliation, yielding a more accurate estimate for each area. Only clinical trial publications were included; reviews, letters, and case reports were excluded. Limitations include the imperfect nature of affiliation-based filtering. A paper is included if any co-author is from northern or southern Italy, even if the main institution is elsewhere. Regional totals may be higher than those reported in earlier all-northern or all-southern Italy queries because these searches use a wider set of keywords (cancer, neoplasm), and papers with co-authors from more than one region are counted in each region. The data show the number of papers with at least one author from that region, not the number of unique papers. Research funding data were drawn from the Italian Association for Cancer Research (AIRC), the Alliance Against Cancer (ACC), and the Italian Ministry of Health’s IRCCS competitive grant records [32,33,34]. Clinical trial density data were drawn from the medical literature. Trial density was calculated as follows: Total Number of Clinical Trials/Total Regional Population × 100,000 inhabitants.

### 2.3. Sample Size and Statistics

There was no predetermined number of papers planned for inclusion in this 5-year analysis. However, a post hoc statistical analysis showed 100% power (beta = 0) with a Type I/Type II alpha error of 0.05 (*p* < 0.00001, which is close to *p* ≈ 0), based on the average number of papers per year/region). The difference between groups: 80.77% − 19.23% = 61.54% (IC 95% (Newcombe method): from 59.30% to 63.71%). We employed a MedCalc Software Ltd. (Ostend, Belgium) power calculator for comparison of means (https://www.medcalc.org/en/calc/power-comparison-of-means.php; Version 23.6.2; accessed on 16 July 2026). This high power remained even when the southern Italy data varied by up to 200%. As a result, the difference is highly reliable, even with a wide range of possible under- or overestimates in the data.

## 3. Results

Table 1 depicts the medical oncology scientific outputs and structural/organizational data split by administrative region. Overall, northern Italy’s scientific output during the 2021–2026 period was 4–6 times higher than that of southern Italy. A balanced, well-constructed search yields an estimate of approximately 2100–3809 publications, representing oncology clinical trials from northern Italy’s major research centers, including IRCCS institutions.

**Figure 1:**
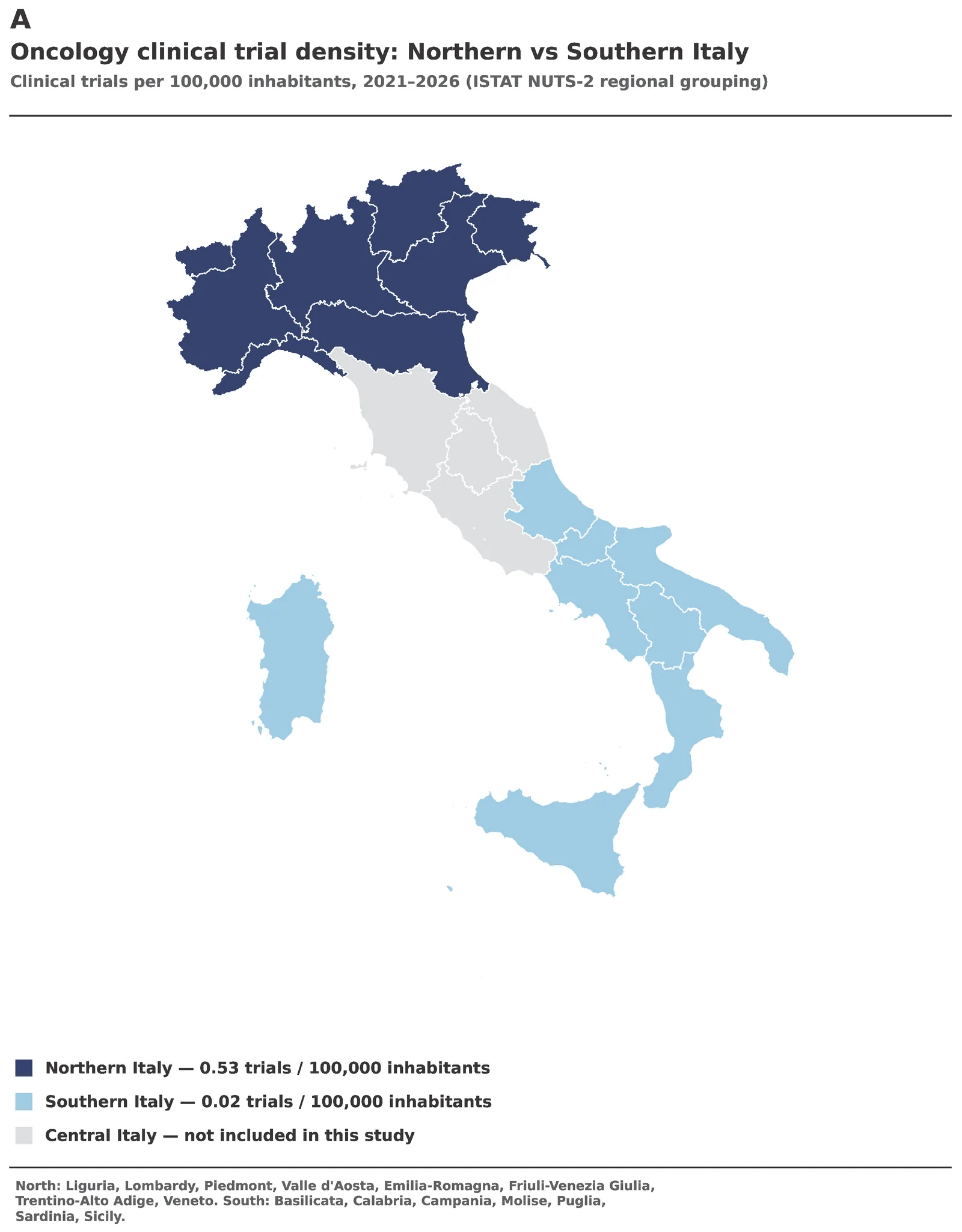

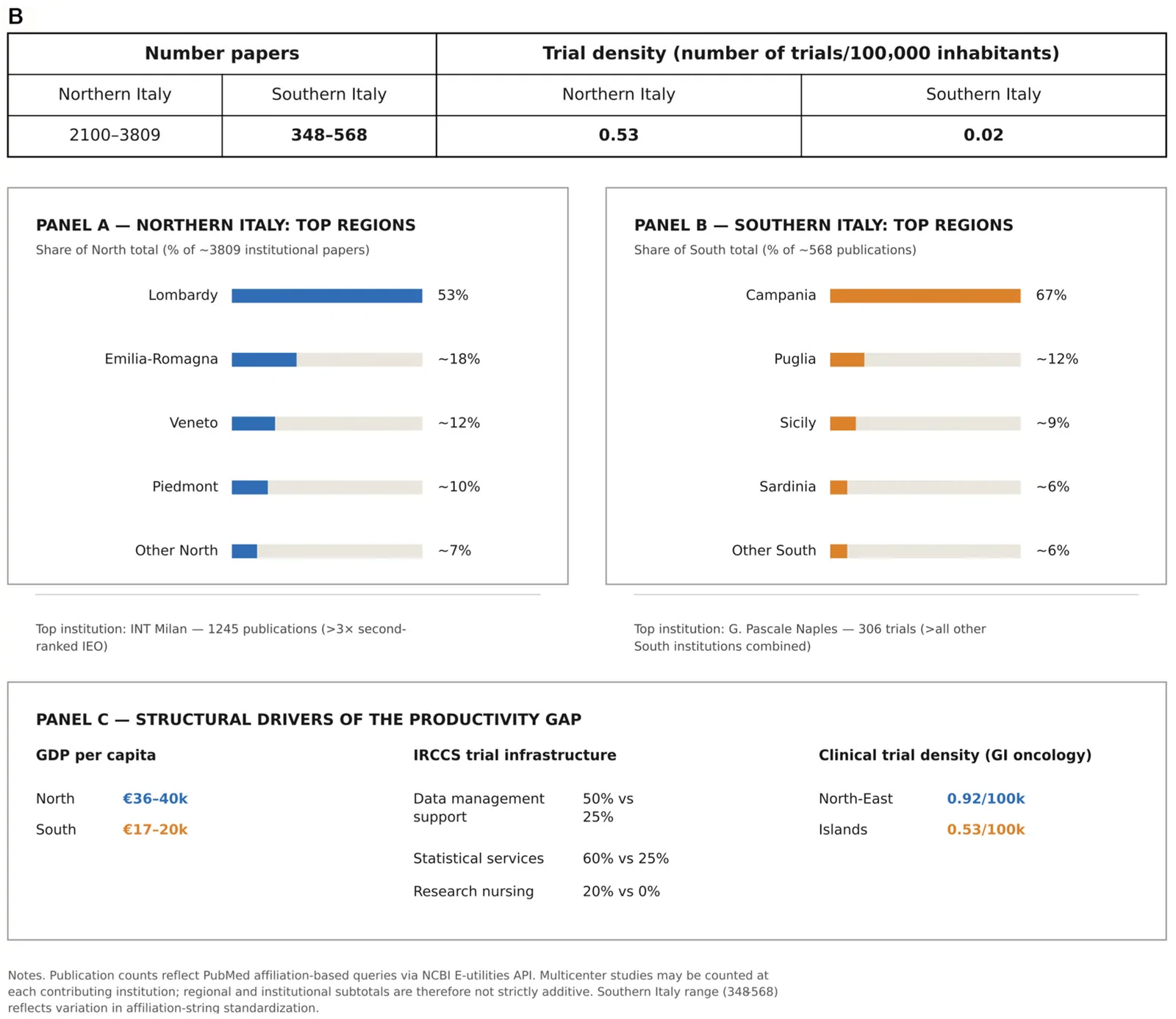
Publication output gaps between northern and southern regions in Italy. (**A**) heath map of oncological trials across Italy (**B**) Number of papers and trial density according to northern (panel A) and southern (panel B) Italy; structural drivers of the productivity gap (panel C).

In total, 739 papers were randomized controlled trials, with Lombardy alone accounting for roughly 53% of all oncology clinical trials in northern Italy. This concentration is driven by the presence of major IRCCS cancer centers in Milan, where INT Milan is the dominant single institution. The top three regions, i.e., Lombardy, Emilia-Romagna, and Veneto, account for roughly 83% of all oncology trial output in northern Italy during the study period. Figure 1 shows the publication output gaps between northern and southern regions in Italy. In northern Italy, Lombardy dominates by a wide margin, driven by Milan’s concentration of world-class oncology institutions, such as the Istituto Europeo di Oncologia-IEO, Fondazione IRCCS Istituto Nazionale Tumori, Humanitas Research Hospital, and the large university hospitals, i.e., Ospedale Niguarda, Istituto San Raffaele, and Policlinico. Emilia-Romagna ranks second, with Bologna (IRCCS Istituto Ortopedico Rizzoli, Policlinico Sant’Orsola), Modena, and Parma—all strong academic medical centers with active oncology research programs. Veneto ranks third, with Padua and Verona as the main contributors, both of which host large university hospitals with internationally active oncology departments. Piedmont is fourth, mainly represented by Turin, which hosts Città della Salute e della Scienza and the Candiolo Cancer Institute, followed by Liguria with the Istituto Nazionale Tumori (INT).

In southern Italy the most reliable estimate may likely fall between 348 and 568, derived from the most comprehensive affiliation-based query using the MeSH term neoplasms and covering all southern Italian regions (Campania, Puglia, Calabria, Sicily/Sicilia, Basilicata, Molise, Sardinia/Sardegna) and their major cities (Naples, Palermo, Bari, Catania, Messina, Cagliari, Salerno, Cosenza, Catanzaro, Lecce, Foggia, Taranto, Potenza, Reggio Calabria) in the author affiliation field. However, a possible caveat is that some papers from southern Italian institutions may use non-standard affiliation strings (e.g., abbreviations or sub-institution names). In southern Italy, Campania is by far the most productive region, accounting for roughly 67% of the macro-area output, reflecting the concentration of major academic and research medical centers in Naples. The IRCCS Fondazione G. Pascale in Naples dominates the entire southern Italy output with 306 trials—more than all other institutions combined. This is the most important dedicated cancer IRCCS in southern Italy and reflects its size and the concentration of affiliated structures around it. The three Campania institutions together account for nearly 447 publications, underscoring the extraordinary concentration of oncology research activity in Naples. Puglia ranks second among regions, powered by both its public university and a cancer-focused IRCCS. Sicily, despite having three active university hospitals, produces fewer registered clinical trials than Puglia, suggesting lower engagement and participation in these trials. Messina comes surprisingly close to Catania despite being a smaller city, driven by the presence of the university hospital. Molise and Basilicata have negligible independent output and likely participate mainly as satellite sites in multicenter trials led by northern or Campanian institutions, with the remarkable exception of the Centro di Riferimento Oncologico della Basilicata IRCSS, which punches well above the region’s population size as a dedicated cancer referral center.

Northern Italy runs circa 74% more oncology trials per capita than southern Italy. The presence of multidisciplinary tumor boards is essential for producing case series, observational research, and trial enrollment. Their distribution mirrors the publication gap: southern Italy has less than half the MDT penetration of northern Italy, a key structural barrier to research participation and publication output. Population-based cancer registries, as well as cancer screenings, generate research cohorts and surveillance data and are the foundation of epidemiological research and secondary prevention. Their uneven distribution further limits southern Italian publication potential. A six-fold gap exists between northern and southern regions in colorectal cancer screening uptake, reflecting both patient access and institutional research readiness.

## 4. Discussion

Italy consistently ranks among the top five European countries for oncology publications, typically behind Germany and the United Kingdom and roughly on par with France [20,35]. When normalized by the number of active oncologists or researchers, Italy has, in some domains, emerged as the country with the largest proportional contribution globally [28]. However, within Italy, this productivity is highly concentrated across regions. Analysis of institutional affiliations in high-impact oncology journals consistently shows that most Italian-affiliated papers originate from institutions headquartered in northern Italy [28,36]. In southern Italy, institutions in Campania represent the outstanding dominant node, but the southern absolute output remains substantially lower than that of their northern counterparts [37]. This north–south disparity is significant and well documented. Italy has been described in the oncology literature as a “differentiated speed” country, a term that specifically refers to the north–south research divide, one of the most studied regional economic disparities in Europe [38,39]. Gross Domestic Product per capita in the northern regions (approximately €36,000–€40,000) is roughly double that of the southern regions (approximately €17,000–€20,000) [40]. Public and private investment in research and development follows a similar pattern: expenditure as a percentage of regional Gross Domestic Product is consistently higher in Lombardy, Emilia-Romagna, and Piedmont than in any southern region [40]. This economic asymmetry shapes university endowments, hospital budgets, research staff hiring, and access to competitive grant opportunities [40,41,42]. It also influences the density of technology companies, the presence of the pharmaceutical industry, and the number of biotech incubators, all of which contribute to the ecosystem supporting clinical and translational oncology research [42].

As depicted in Figure 1, northern Italy produces an estimated 4–6-fold more oncology research publications than southern Italy, a difference driven by several factors, including a greater concentration of research-focused IRCCS institutes, better-funded university hospitals, higher organizational levels, such as more multidisciplinary teams, and greater access to clinical trials and infrastructure [43]. The COVID-19 pandemic from 2020 onward significantly disrupted oncology research across Italy, but its effects were not geographically uniform [44]. The COVID-19 emergency highlighted the vulnerabilities of southern institutions: lower baseline intensive care unit capacity, fewer digital infrastructure resources for telemedicine, higher rates of trial suspension, and greater difficulty maintaining continuity of research staff during lockdown periods [44,45]. These emergency-related disruptions further widened a pre-existing gap.

The IRCCS centers form the backbone of Italian cancer research and collectively produce most high-impact oncology publications [46,47]. Among IRCCS centers, ten are designated as pure oncology institutes. The distribution of specialized cancer centers, particularly the IRCCS networks, is markedly skewed toward the north and center of the country, creating a critically uneven geographic distribution [46,47,48].

This concentration of elite research infrastructure in northern cities directly shapes publication productivity, as IRCCS centers consistently demonstrate higher research engagement, better resource availability (data management, statistical services, dedicated research nurses), and greater faculty-level commitment to scientific output [48,49]. Critically, IRCCS centers reported significantly better support infrastructure for trials than non-IRCCS centers: 50% vs. 25% for data management services; 60% vs. 25% for clinical statistics services; and 20% vs. 0% for dedicated scientific nursing staff. Since IRCCS centers are disproportionately located in the north, the resource advantage compounds the geographic imbalance [48,49]. The IRCCSs present in Lombardy, Piedmont, and Veneto account for a strikingly large share of Italy’s total oncology publication output. For instance, the IRCCS centers in Turin has published more than 1200 articles in international journals over seven years, accumulating more than 13,000 citations—metrics that few southern institutions can match cumulatively [50]. The Nature Index, which tracks contributions to high-quality natural science journals, consistently lists northern Italian cancer institutes among Italy’s top-performing research institutions, with no southern oncology institute appearing among the national leaders in any recent ranking period [51]. Disparities vary somewhat by oncology subspecialty. In gastrointestinal oncology, radiation oncology, transplants, and immuno-oncology, northern institutions maintain a commanding lead in output volume. In areas with lower technological infrastructure requirements—such as palliative care research, cancer communication, and health inequities research—southern institutions show more proportional engagement, though absolute numbers remain low.

**Figure 2:**
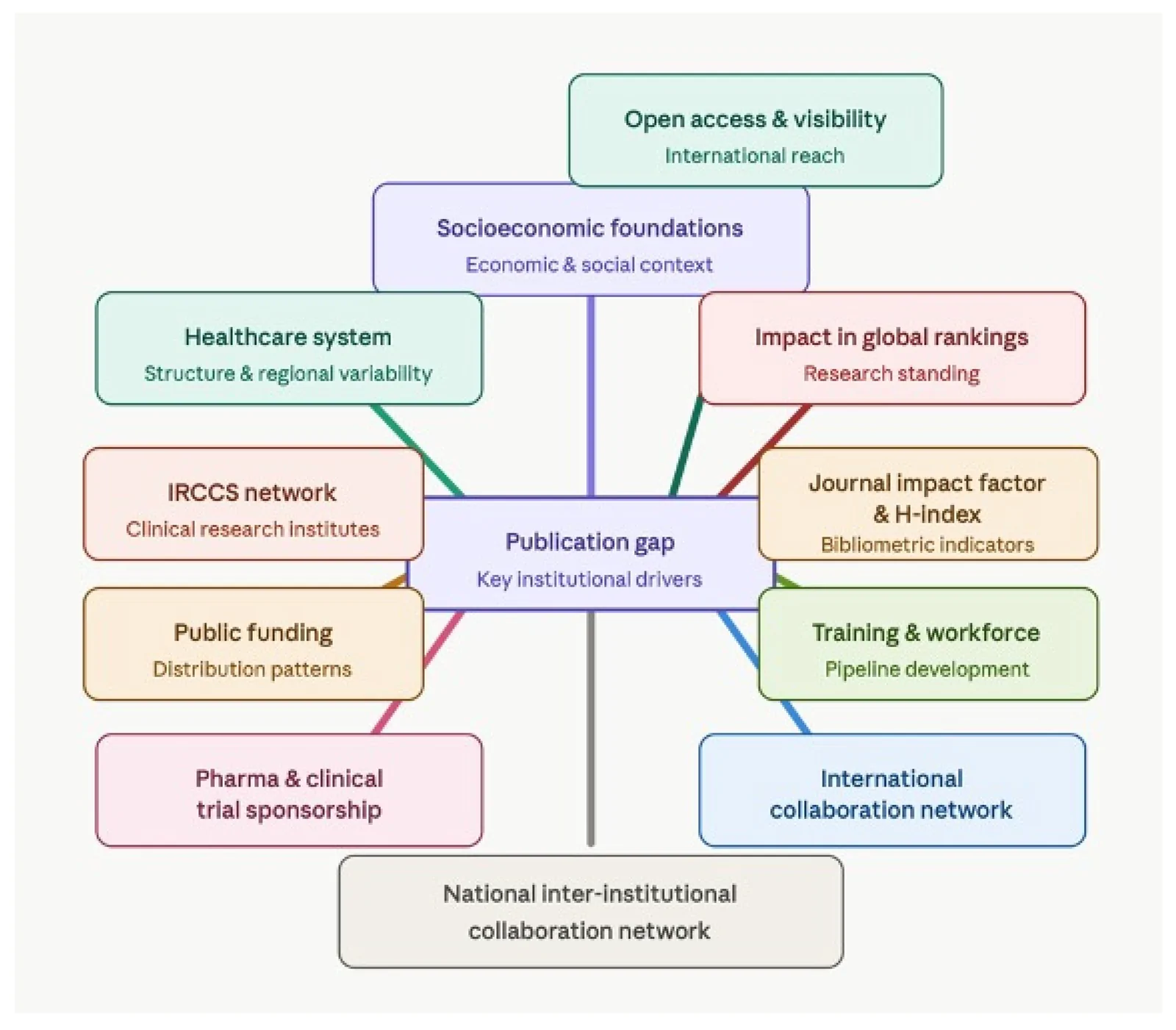
Key factors influencing publication output.

As shown in Figure 2, the key drivers of the publication gap are multiple. The disparity is primarily structural. The north houses most Italy’s designated cancer IRCCS centers, which have dedicated research mandates and funding streams [43]. The Italian National Health Service is organized at the regional level, with each region responsible for healthcare planning, funding allocation, and service delivery. This decentralized structure, while intended to promote local responsiveness, has, in practice, produced wide variation in healthcare quality, efficiency, and investment capacity. Multiple studies have documented significant disparities in cancer care quality across Italian regions [28,36,49]. Regional inequalities in cancer care persist and can affect survival, with patients in the southern regions facing longer waiting times, reduced access to innovative therapies, and reduced access to multidisciplinary oncology teams [52].

The Italian Ministry of Health allocates competitive research grants to IRCCS institutions through the “Ricerca Corrente” (ongoing research) and “Ricerca Finalizzata” (targeted research) programs [53]. While the formal allocation criteria are merit-based, institutional track record, publication history, and existing infrastructure, which mostly favor established northern centers, therefore play significant roles in competitive success.

Southern IRCCS centers, operating under tighter regional budgets and with smaller research faculties, face structural disadvantages in these competitions. The Fondation AIRC, Italy’s primary private funding body for cancer research, similarly channels most of its investigator grants to institutions in northern and central Italy [32]. The geographic distribution of AIRC-funded projects mirrors the broader publication and institutional resource disparities, with Lombardy alone accounting for a plurality of funded projects in the most recent grant cycles [32].

Industry-sponsored clinical trials—which represent a substantial share of clinical oncology research and often result in co-authorship publications—are heavily concentrated in northern Italy [54]. The presence of major pharmaceutical companies, contract research organizations (CROs), and regional regulatory networks in the Milan metropolitan area and surrounding regions creates a gravitational pull for industry-sponsored trial sites [54]. Polignano et al. recently quantified disparities in the availability of gastrointestinal oncology trials, finding that clinical trial density was 0.92 trials per 100,000 inhabitants in the northeast versus 0.53 per 100,000 in the islands, a nearly two-fold difference, with the south and center areas showing intermediate values [28].

The ACC, established in 2002 under the Italian Ministry of Health, provides a formal framework for the National inter-institutional collaboration network [33,47]. The ACC has been instrumental in establishing shared biobanks, genomics platforms, and collaborative research projects. However, participation intensity within the ACC is uneven: northern institutions lead most collaborative research programs, while southern IRCCS centers more often play peripheral roles in multi-site studies. The Federation of Italian Cooperative Oncology Groups (FICOG), established in 2015 with 17 cooperative groups, likewise shows geographic concentration [55]. The academic and administrative leadership of most FICOG member groups is based in northern institutions, which translates into headquarters effects on publication authorship, the centrality of investigator meetings, and the location of data analysis sites [55]. Moreover, Italian oncology’s international reputation is closely tied to a small number of institutions with deep-rooted international networks, for instance, IRCSS centers in Milan, Rome, and Naples, and academic departments at the Universities of Bologna, Padua, and Turin. These institutions maintain longstanding collaborative ties with the EORTC, ESMO, and ASCO cooperative groups, as well as leading European and American cancer centers [48]. Even if their network centrality metrics are consistently lower, southern Italian institutions are not absent from international collaboration. The IRCSS in Naples, for example, has contributed meaningfully to European cooperative trials in melanoma, lung cancer, and gastrointestinal oncology [37]. In bibliometric network analyses, southern Italian oncology nodes appear in peripheral rather than hub positions, receiving fewer collaborative citations and contributing less frequently to first- and last-authorship positions in high-impact multinational papers [56].

The training environment for medical oncology trainees also diverges significantly between the north and the south [57]. A survey conducted by the Italian Association of Medical Oncology found that while most oncology residents across Italy engage in research activities, those training in northern institutions report significantly more exposure to clinical trial participation, manuscript preparation with senior mentorship, and conference presentation opportunities [57]. This training gap perpetuates the productivity divide into the next generation of researchers [42].

Citation impact broadly mirrors publication volume. Northern Italian oncology institutions demonstrate higher aggregate h-indices, higher average journal impact factors for their publications, and more frequent citation in systematic reviews and clinical guidelines [58]. When Italian oncology is collectively cited by international meta-analyses and guidelines, as frequently occurs in the context of landmark European cooperative group trials, the cited institutions are disproportionately located in the north [58]. The gap also impacts institutions’ global rankings. U.S. News Best Global Universities rankings in oncology consistently place several northern Italian institutions, i.e., the Universities of Milan, Bologna, and Padua) in the top tiers of European research universities [59]. No southern Italian university appears in the leading oncology research university rankings on this or similar platforms. This gap reflects both publication volume and citation-weighted quality metrics that define such rankings.

The adoption of open-access publishing has been broadly uniform across Italian regions, as it is largely driven by journal-level policies rather than institutional location. However, the ability to pay article processing charges (APCs), which can range from €1000 to over €3000 per article in top journals, systematically disadvantages institutions with smaller research budgets, which are more prevalent in the south [60]. This financial barrier can limit the international visibility of southern Italian oncology research even when the science is meritorious.

The findings presented here converge to paint a consistent picture: northern Italy substantially outperforms southern Italy in medical oncology research productivity across all measured dimensions. This is not primarily a reflection of individual researchers’ quality or scientific ambition in the south, but of structural, financial, and, primarily, organizational conditions that systematically advantage northern institutions. Notably, several of the top medical oncology researchers in northern Italy came from the south. The mechanisms driving this disparity are interconnected and mutually reinforcing. Economic prosperity in the north enables larger hospital budgets, more research staff, and greater institutional capacity to compete for grants. A denser pharmaceutical ecosystem creates more opportunities for industry-trial collaborations. Established IRCCS centers attract talented researchers through competitive salaries and infrastructure, further concentrating human capital. International collaboration networks, once formed, tend to reproduce themselves. Prestigious northern institutions attract collaborators who, in turn, co-author papers that enhance the northern institutions’ h-index and visibility, creating a cumulative advantage.

This paper has several limitations due to its narrative nature and warrants some consideration. First, a major limitation is represented by the lack of univocal and globally accepted measure of scientific productivity [61,62,63]. Secondly, the absence of a standardized national database linking individual publications to precise regional affiliations represents a significant methodological constraint. Some institutions, particularly those co-affiliated with universities and hospitals across different regions, create attribution ambiguity [64]. Additionally, the present analysis is narrative in nature; formal meta-analysis of bibliometric data was not performed. Paper counts can vary depending on the quality of PubMed affiliation indexing [65]. Some multicenter trials may list only one center. For a definitive systematic review, a direct PubMed Advanced Search with a manually curated query is recommended. Some trials appear across multiple institutions, as occurs in multicenter studies, so regional and institutional totals are not strictly additive. Counts reflect PubMed affiliation indexing via the NCBI E-utilities API [66]. Secondly, the data presented are not to be interpreted as a ranking of merit. Indeed, institution-level counts overlap: a paper with co-authors from multiple institutions is counted at each institution, so institution subtotals exceed regional totals. On the other hand, some institutions may report a lower count, reflecting a specialty focus on musculoskeletal oncology rather than broad medical oncology, such as the orthopedic-focused Rizzoli Center in Bologna. Similar considerations apply to the National Institute of Gastroenterology IRCCS “Saverio de Bellis” in Castellana Grotte, Italy, dedicated to gastrointestinal diseases, and to the Mediterranean Institute for Transplantation and Advanced Specialized Therapies IRCCS ISMETT in Palermo, focused on transplantation [67,68]. It is important to note that the absolute output of southern Italian medical oncologists is not negligible. The Sicilian 5-year study yielded 283 oncology papers with a combined impact factor of ~1014, from units often overlooked in national analyses [44,69]. However, the National Cancer Institute of Milan only yielded an impact factor of 6622.10 over the same time span. The gap is therefore one of scale and infrastructure, not of talent. A critical distinction must be made: the data do not suggest that southern Italian medical oncologists are less scientifically capable or less motivated. When southern IRCCS centers are assessed relative to their available resources—in terms of per-researcher publication rates or publication rates adjusted for institutional size—the gap with the north narrows considerably. The primary driver of the aggregate disparity is structural: fewer resources, fewer research-dedicated positions, less infrastructure, and less industry engagement. This distinction is important because it points toward addressable policy solutions rather than immutable regional characteristics. The IRCCS in Naples provides an illustrative example. Despite the structural disadvantages of its southern context, it has maintained and significantly expanded a nationally and internationally recognized research profile across several oncology subspecialties, participated in cooperative clinical trials, and contributed meaningfully to the Italian oncology literature [70]. Its success demonstrates that southern institutions can achieve research excellence but require commensurate structural and organizational support to do so at scale.

The geographic concentration of medical oncology research has direct consequences for the quality and representativeness of clinical evidence [71]. Clinical trials conducted predominantly in northern Italy may enroll patient populations that systematically differ from those in the south in comorbidity profiles, lifestyle factors, genetic ancestry, and socioeconomic determinants of health. If southern Italian patients are under-represented in clinical trials—as the trial density data strongly suggest—the resulting evidence base may be less applicable to their care, potentially contributing to survival disparities already documented in the literature [72,73]. Moreover, concentrating first authorship and investigator roles in northern institutions concentrates scientific agenda-setting power in those regions. Research priorities—which cancer types to study, which endpoints to pursue, which populations to focus on—are inevitably shaped by the institutions and researchers leading trials and writing grant applications. A more geographically balanced research enterprise would enrich this agenda.

This analysis presents several potential policy implications, summarized in Table 2 along with tentative goals. New directions may include funding rebalancing, decentralized trial models, inter-regional mentorship and twinning programs, digital research infrastructure investment, and alignment of Italy’s national research evaluation system assessments.

## 5. Conclusions

Scientific publication productivity in Italian medical oncology is marked by a significant and persistent north–south divide. Northern institutions lead in publication volume, citation impact, clinical trial density, research funding, and centrality within international collaboration networks. Southern institutions, while individually capable of research excellence, face structural disadvantages that constrain their aggregate contribution to the national and global oncology evidence base. This disparity is not an inevitable feature of Italian geography or culture. It is the product of decades of differential investment, institutional development, and policy choices that have concentrated scientific resources in the north. The consequence is a research landscape that under-represents the cancer burden, patient populations, and scientific talent of southern Italy, with direct implications for equitable evidence generation and patient care. Addressing this divide requires a multi-pronged policy response: targeted funding mechanisms, decentralized research infrastructure, structured inter-institutional mentorship, and evaluation frameworks that recognize contextual productivity. Italy’s continued growth as a global leader in oncology research depends, in part, on realizing the full scientific potential of all its regions.

## Figures and Tables

**Table 1:** Medical oncology scientific outputs and structural/organizational data split by administrative region. Results from querying the PubMed NCBI database directly via the E-utilities API MeSH: neoplasms/publication type: clinical trial/affiliation-based search (January 2021–March 2026, https://eutils.ncbi.nlm.nih.gov/entrez/eutils/esearch.fcgi?db=pubmed&retmax=100&retmode=json&term=Neoplasms%5BMeSH+Terms%5D+AND+Clinic (accessed on 1 June 2026)).

Indicator	Northern Italy	Southern Italy
Estimated oncology publications	~2000–3809	~300–500
Relative output multiplier	Baseline (1×)	~0.15–0.25×
Clinical trials per 100,000 inhabitants	0.53	0.02
Relative publication output	4–6 fold higher	Baseline
Combined impact factor	6622.10 ^$^	1014.6 ^$^
Trial center share (national)	~58%	~25%
Share of national cases covered	46% of cases registered	24.7% *
Per capita trial rate vs. national avg.	+38% above average	−42% below average
Multidisciplinary tumor boards coverage	73.3%	32.3%
IRCCS Cancer Institute distribution	8 out of 10 major IRCCS	1 major IRCSS + 2 minor ^
Trial center geographic clustering	High overlap/dense	Sparce/isolated
Colorectal screening gap	High uptake	~6 fold lower than north

^$^ Data refer to National cancer Institute of Milan, Lombardy, and the Sicilian output. * Low coverage also limits research data quality. ^ Fondazione Pascale, Naples, plus two smaller ones in Bari and Rionero respectively.

**Table 2:** Policy implications reported according to theme, along with tentative goals.

Policy Area	Theme	Description	Goal
Funding rebalancing	Equity	Targeted grant mechanisms for southern and island institutions, modeled on EU structural fund approaches, with evaluation criteria that account for contextual constraints.	Offset baseline disadvantages of under-represented regions
Decentralized trial models	Access	Adoption of hybrid and decentralized clinical trial designs, building on pandemic-era regulatory flexibility, to extend trial access to southern sites without requiring full IRCCS infrastructure.	Broaden geographic reach of clinical research participation
Inter-regional mentorship and twinning	Capacity	Formal partnerships between northern and southern oncology institutions structured around shared projects, mentorship of southern trainees, and joint grant applications.	Accelerate capacity building in the south while enriching northern institutions
Digital research infrastructure	Access	Investment in bioinformatics platforms, biobank networks, and digital data management systems accessible to all IRCCS and academic oncology centers regardless of location.	Reduce infrastructure gap at relatively low cost
ANVUR assessment alignment	Evaluation	Reform of Italy’s VQR/ANVUR research evaluation system to incorporate contextual productivity metrics that account for institutional resource levels.	Enable more equitable assessment of southern researchers’ contributions

## Data Availability

These data were derived from the following resources available in the public domain: [PubMed: https://pubmed.ncbi.nlm.nih.gov (accessed on 1 June 2026)].

## Abbreviations

The following abbreviations are used in this manuscript:

IRCCS	The Oncological Scientific Institutes for Hospitalization and Care (Istituti di Ricovero e Cura a Carattere Scientifico)
CCC	Comprehensive Cancer Center
OECI	Organization of European Cancer Institutes
AIRC	Italian Association for Cancer Research
ACC	Alliance Against Cancer
FICOG	Federation of Italian Cooperative Oncology Groups
